# Vagus Nerve Stimulation in Alzheimer’s Disease and Mild Cognitive Impairment: Current Evidence and Future Directions

**DOI:** 10.3390/neurosci7030052

**Published:** 2026-04-27

**Authors:** Ruth Narramore, Mudasar Aziz, Sheharyar Baig, Joyce S. Balami, Arshad Majid, Ali N. Ali

**Affiliations:** 1 Sheffield Teaching Hospitals NHS Foundation Trust, Sheffield S10 2JF, UK; mudasar.aziz@nhs.net (M.A.); arshad.majid@sheffield.ac.uk (A.M.); ali.ali@sheffield.ac.uk (A.N.A.); 2 Department of Neuroscience, Sheffield Institute for Translational Neuroscience, University of Sheffield, Sheffield S10 2HQ, UK; sbaig1@sheffield.ac.uk

**Keywords:** vagus nerve stimulation, Alzheimer’s disease, mild cognitive impairment, cognition

## Abstract

Vagus nerve stimulation (VNS) may improve cognition and promote underlying brain health through various mechanisms including the noradrenaline and cholinergic pathways. Whilst early human studies used invasive devices (iVNS), recent decades have seen the emergence of non-invasive devices that stimulate the vagus nerve transcutaneously (tVNS) via either the cervical branches in the neck (tcVNS) or the auricular branch in the ear (taVNS). With this increase in more accessible devices, tVNS is gaining interest as a novel therapy in mild cognitive impairment (MCI) and Alzheimer’s disease (AD). This targeted review aims to understand the current evidence in human trials in this specific population. PubMed, Cochrane, EMBASE, MEDLINE, and Google Scholar were searched. Six human interventional studies were found (one iVNS; five taVNS). VNS is well tolerated and study designs demonstrate feasibility within this population for future blinded and appropriately powered long-term studies with participants applying tVNS at home. However, protocols and tVNS settings remain variable. Working memory domains such as verbal fluency and 3D processing show the most promise but global cognitive scores were also sensitive in some cases. The role of biomarkers of tVNS activity and its effect on AD markers and neuroinflammation should be considered in the design of future studies.

## 1. Introduction

Alzheimer’s disease (AD) is the most common form of dementia and is associated with a high burden of morbidity and mortality [1]. Mild cognitive impairment (MCI) describes impaired cognition disproportionate to age. Around a third of people with MCI progress to AD. [2] Multiple approaches to treatments are under investigation, but whilst several have shown promise, and a few are now entering clinical practice in some countries, there is not yet a single globally agreed approach to a disease-modifying therapy. This is likely, in part, due to the complexity of the disease model of Alzheimer’s and interactions between genetics, the environment, metabolism and psychosocial factors [1].

The vagus nerve (VN) connects various body systems to the central nervous system through afferent and efferent pathways with multiple roles in health and pathology. Vagus nerve stimulation (VNS) via an implantable stimulator is an approved therapy for refractory epilepsy and depression and upper limb motor recovery in ischaemic stroke. Non-invasive transcutaneous VNS (tVNS) can be delivered to the auricular branch of the vagus nerve in the ear or cervical branch in the neck [3].

The use of tVNS in cognitive impairment is a relatively new area of interest in research and there are several proposed mechanisms of action largely derived from animal models that have yet to be proven in human clinical studies. The VN communicates to multiple areas of the brain via the Locus Coeruleus, (LC) including the amygdala, hippocampus, prefrontal cortex and thalamus, utilising the neurotransmitter noradrenaline (NA). The accumulation of amyloid and tau in the LC is an early pathological feature preceding clinically detectable memory impairment by decades. Studies have demonstrated activation of the LC-NA pathway can promote neuroplasticity, reduce the accumulation of pathological proteins and promote protective microglial function such as phagocytosis and clearance of amyloid-beta (Aβ), which could prevent or even reduce the formation of damaging plaques. Conversely, damage to the LC, and subsequent failure of the NA system and spread of pathological amyloid and tau along its network, disrupts and suppresses the cholinergic pathway until eventually neurones become damaged, following which overt memory impairment manifests. The VN also projects to the nucleus tractus solitarius (NTS) which relays to the nucleus basalis of Meynert, another area damaged in early stages of pre-clinical disease. The nucleus basalis is a key cholinergic source to the cortex and is implicated in AD progression [4]. Therefore, VNS provides a potential therapy that can protect against the progression of Alzheimer’s disease at its earliest stage.

**Figure 1:**
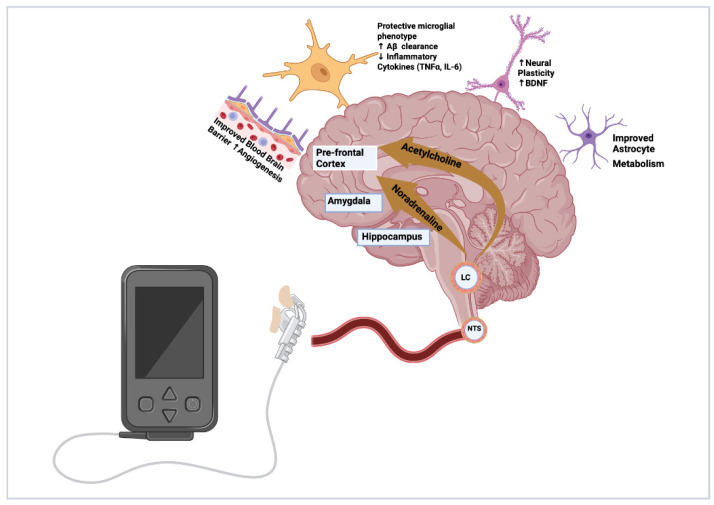
Mechanisms of action by which VNS promotes a healthier brain microenvironment and protects against AD pathology. Figure created by Sheharyar Baig. Created in BioRender. Aziz, M. (2026) https://BioRender.com/c7tvrzs—accessed on 24 January 2026.

Additionally, through both the NA and in particular the cholinergic pathway, the VN modulates local and systemic inflammation. VNS has been investigated as a treatment in other inflammatory conditions such as inflammatory bowel disease (IBD) through its systemic anti-inflammatory role via its efferent pathways to the gut and spleen [5]. This anti-inflammatory process is potentially neuroprotective in several ways including suppressing inflammatory cytokines, promoting a neuroprotective phenotype in microglia, improving blood–brain barrier (BBB) function, promoting a less reactive and improved metabolic profile in astrocytes, and improving cerebral blood flow and microvascular health [6,7,8,9,10] (Figure 1).

tVNS devices are now widely available and produced by multiple companies for commercial and medical use at relatively low cost. Devices are increasingly user-friendly and can be applied by the user with minimal training, making them feasible in an MCI or early AD population. They offer the advantage of a non-pharmacological adjunct with minimal side effects or medication interactions.

Whilst there are several general reviews of VNS, the authors aim to highlight that to date, there have been very few human clinical trials in the MCI and AD population. The aim of this review is to synthesise outcomes from published clinical trials and mechanistic studies in this specific group, both to highlight the knowledge gaps and explore any emerging themes in the evidence that can be used to improve the efficacy and output of future studies.

## 2. Materials and Methods

**Figure 2:**
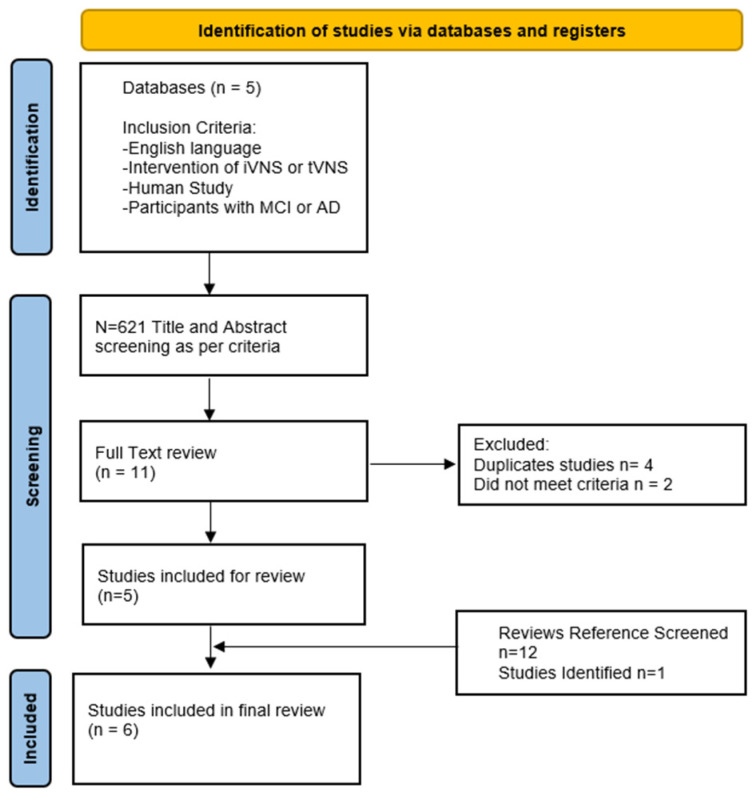
PRISMA flow chart showing selection process for studies [17].

The databases searched between 1 August 2025 and 1 April 2026 were PubMed, Cochrane, Google Scholar, MEDLINE and EMBASE using the terms: “VNS” OR “Vagal Nerve Stimulation” OR “Vagus Nerve Stimulation”[Mesh] AND “Alzheimer’s Disease” OR “Alzheimer Disease” OR “Mild cognitive impairment” OR “Dementia” OR “Alzheimer Disease”[Mesh] OR “Cognitive Dysfunction”[Mesh] OR “Cognitive Impairment” OR “Cognition” OR “MCI” OR “Cognitive Impairment”. Only English Language Studies were included. Search terms were devised in consultation with experts within the research group. Titles and abstracts were screened for relevant human RCTs, single-arm interventional studies and mechanistic studies. The references of several reviews identified during title screening were also screened for further articles [3,4,6,7,11,12,13,14,15,16]. Studies were included if they evaluated the effects of VNS in patients with MCI or AD utilising different study designs. We included all studies irrespective of their primary or secondary outcomes. The initial search results were reviewed by 2 authors (RN and AA) and duplicate and irrelevant articles were removed after screening titles and abstracts. Full texts were then reviewed for final inclusion; 9 clinical manuscripts were initially identified. However, on further review, 4 were found to be duplicate studies. A further clinical study was found via reference searches, giving a total of 6 clinical trials which were finally included (Figure 2).

The small number of studies had variable study designs; therefore, data was extracted according to Cochrane narrative synthesis guidance rather than meta-analysis. Available data on protocols, participant population, and methods of measurement including cognitive measures were extracted and tabulated. These data were then checked by a second author (MA) and any discrepancies were reviewed and resolved. As this review was partly to demonstrate knowledge gaps, studies with missing data were included and any gaps were recorded.

Bias was measured using the Mixed Methods Appraisal Tool (MMAT) which is a critical appraisal instrument designed for systematic mixed-studies reviews, enabling consistent assessment of qualitative, quantitative, and mixed methods designs within a single framework. It contains five methodological domains: qualitative research, randomised controlled trials, non-randomised studies, quantitative descriptive studies, and mixed methods studies. Users first confirm that a study is empirical, then select the appropriate domain, each with five design-specific criteria. The author (RN) rated each criteria as “Yes” or “No” as per the MMAT guidance, generating a score out of five, where higher numbers indicate less bias (e.g., Yes = 1 point) [18]. A breakdown of scores is available on request. This allowed for a descriptive comparison between studies, emerging themes and limitations. We undertook a narrative synthesis using the Cochrane Consumers and Communication Review Group framework [19].

To identify ongoing studies of VNS in MCI/AD, clinicaltrials.gov was searched with search terms Alzheimer’s Disease and VNS (10 February 2026). Further, our discussion utilised the wider VNS literature to help understand pathophysiological principles and gaps in the current evidence base.

## 3. Results

Only six interventional studies were identified that specifically used VNS in an MCI or AD population [20,21,22,23,24,25]. They differed greatly in their populations, interventions and protocols. Most studies were small (five of six studies had ≤60 participants) and designed towards safety, feasibility and mechanisms rather than treatment effect, although Wang et al. specifically powered their study to detect differences in MOCA-B [20]. There were only two longitudinal studies of VNS in MCI/AD—one single-arm study with invasive VNS and one RCT with tVNS [20,25]. Three studies focused on non-cognitive outcomes using functional MRI (fMRI) and vagus somatosensory evoked potentials (VSEPs) as biomarkers of VNS activity [21,22,23].

All tVNS studies used auricular rather than cervical application with near equal split between tragus and conchae. They demonstrated feasible options for blinding. Wang et al. achieved double blinding of the acupuncturist applying the device. More significantly, both Dolphin et al. and Wang et al. demonstrated that, with only a short duration of training, participants with MCI would be able to feasibly self-apply tVNS at home for longer periods [20,24]. This has been achieved in other studies with patients with normal cognition [3]. Wang et al. described a model to support compliance using electronic diaries checked remotely once a month by investigators and a video call to check technique once a week, but did not report their compliance data for this technique (Table 1).

There were a variety of VNS device settings. Murphy and Metzger and Polak used tragal application and found intensities ranging 7–8 mA were well tolerated. Dolphin and Wang used conchae application and found 2–3 mA and 1mA were tolerated respectively. A 20 Hz frequency was used in three of five studies and is common for many devices [3]. Sham stimulation in these studies involved stimulation with the same intensity and duration as the active arm but at an anatomical site away from the area of branches of the vagal nerve. Sham sites in this study included the helix and ear lobe, both well established in previous trials (Table 2).

Across all studies, invasive and transcutaneous VNS delivery was well tolerated with only one patient dropping out due to tolerability across all studies.

The diagnosis of MCI and AD was reached by a variety of protocols largely reflecting local practice. Typically, these included multiple internationally validated cognitive scores and consensus diagnosis tools. Only Merrill used a single cognitive score (MMSE.) Imaging, either CT or MRI, was available for all patients but was typically used to rule out alternative diagnoses including overlapping diagnoses such as vascular disease. No study used more advanced AD-specific imaging such as PET of CSF biomarkers as criteria to recruit patients. This reflects clinical practice and in AD is likely to have less impact on study noise. However, MCI may reflect a variety of aetiologies and specific screening for AD biomarkers may help define a truly AD pathological subpopulation for target intervention. This must be balanced against the prohibitive expense of advanced imaging and patient cost of invasive CSF testing.

Merrill et al. collected serial CSF biomarkers at baseline and 3 and 12 months. CSF tau level varied at baseline (99–764 pg/mL) and there was no statistically significant difference at 3 and 12 months. There was similarly no difference in β amyloid or Aβ42. There was a small but significant increase in phospho-tau compared to baseline (*p* = 0.04) [25]. Dolphin et al. collected CSF biomarkers for sub-analysis but not for all patients. A total of 75% of those tested had positive biomarkers at entry [23].

Only the Wang study was double-blinded. Considering the placebo effect, their primary outcome measure of MOCA-B found no statistically significant improvement in the sham arm. There was significant improvement in both the intervention and sham arms for the secondary outcomes of shape trails test B and the Boston Naming test and no between-group differences. This could reflect either a placebo effect of sham VNS or highlight the vulnerability of some cognitive testing to a “practice” effect. Interestingly improvements in sleep and functional scores in both groups are also suggestive of a placebo effect. Whilst not directly comparable, the invasive Merrill study also noted a modestly improved MMSE in the initial month after implantation before the device was activated. Of the other two single-blinded studies, Murphy et al. did not measure cognition and Dolphin et al. has not yet published in full.

The longest study by Merrill et al. observed more positive results in the first six months of treatment, followed by diminishing results at one year. This could either reflect a weaker treatment affect due to progression of underlying disease or a “wearing off” affect in VNS [25]. It is therefore important for future studies to be of a long enough period to further explore this.

Wang et al. delivered the best-quality study design for blinding and longitudinal data collection and showed significant improvements of 3.2 points in the MOCA-B score at 6 months in the tVNS group compared to 0.3 points in the sham group (*p* = 0.033) [19].

A variety of cognitive scores measured both global cognition and specific cognitive domains. Interestingly, it was the global tests of ADAS-Cog, MMSE and MOCA-B where the most positive results were seen (Table 3). This may be because these were only used in the two longitudinal studies and reflect an effect of longer term tVNS treatment. It would seem unrealistic that short single-intervention studies would lead to changes in global cognitive scores. However, these global tests also generate data across a variety of domains and may be more likely to detect changes across more than one cognitive domain. They also have the advantage of being able to reflect a subjective improvement in quality of life for participants and are widely used across dementia trials, thereby making studies more comparable.

Tests on subgroups of memory were less consistent with a suggestion of improvement in memory recall and 3D navigation [20,24] (Table 3).

Merrill and Wang were the only studies to report on functional outcomes (Clinical Global Impressions Scale and Functional Activities Score respectively.) These are more subjective and therefore less likely to detect change. Whilst Wang did not observe a difference between groups, Merrill showed that at one year, seven participants had maintained and five had improved, although in the absence of a control group, this is hard to interpret [24]. Wang et al. also assessed multiple measures of sleep and found improvements in both tVNS and sham arms [20,25].

An area of further consideration includes concurrent use of cognitive-enhancing medications with either cholinesterase inhibitors (ChEIs) or memantine. No study excluded patients on these medications and as most patients with a diagnosis of AD commence ChEIs, this would be limiting to recruitment. Merrill et al. specifically note that ChEIs may have a synergistic role with VNS. They ensured participants were on stable medication regimes 8 weeks prior to study start but did not stop people starting ChEIs during the study. A total of 10 of 17 participants were on ChEIs at the start of the experiment (mean 45 weeks before), 2 commenced them during the experiment and one increased their dose. The small numbers preclude subgroup analyses, but the stability of treatment prior to enrolment is unlikely to influence their study’s results [24]. The only other study to mention concurrent therapy was Metzger et al., where 11 of 13 of their participants were on anti-dementia drugs. They acknowledged uncertainty about how these drugs may influence VNS effects on VSEP [22]. Polak et al. discussed the confounding effects of SSRIs in their depression arm but not of ChEIs in their AD arm and did not collect this information. The other three studies were all in an MCI population only and did not mention use of cognitive medications, probably because these are not used in routine practice in an MCI population. Future long-term studies would need to consider if a patient were to start these medications during the study and how this might affect their results.

A variety of other outcome measures were considered in these studies. Polak et al. demonstrated significantly longer VSEP latencies following VNS in MCI compared to healthy matched controls, a result not reflected in [22]. Supporting this, Metzger et al. found increasing latencies over pathological groups from healthy to MCI to AD [23]. Murphy et al. found tVNS significantly altered connectivity as seen on fMRI in key areas of memory of the temporal and parietal lobes [21]. In a search of the clinicaltrial.gov registry of current ongoing trials in VNS and cognition, several are listed with a variety of intervention types, time periods, protocols and outcomes. Three studies are in older adults, one in MCI and two in AD (Table 4).

## 4. Discussion

This review synthesises the available clinical and mechanistic evidence for VNS in MCI and AD. Despite widespread accessibility of tVNS devices, the urgent clinical need for disease-modifying therapies, and a theoretical rationale for the application of tVNS in MCI/AD, there are only six interventional human studies. Most of these studies were small and short-term or mechanistically focused rather than focused on clinical efficacy. Study protocols varied to such a degree that making specific between-study comparisons was difficult. This discussion will focus more broadly on any useful emerging themes and important and consistent gaps.

Across studies, VNS was safe and generally well tolerated where reported. Most notably, Wang et al. were able to demonstrate that participants with MCI were able to self-deliver the intervention with minimal training, although the training was delivered by a qualified acupuncturist. As the only long-term tVNS study, they were also the only study to consider compliance. They utilised an online diary and messaging system to facilitate use, although they did not specifically report their compliance data. The single-intervention studies often use glue or adhesive to apply the tVNS probe which requires specialist knowledge of anatomy and can result in variability due to poor adhesion or side effects due to skin irritation. However, modern devices such as those used by Wang et al. and Dolphin et al. are designed to be user-friendly and fit to the ear without adhesive, similarly to an earphone, thus allowing for easy and consistent application by the participant. The disadvantage is that commercially available devices are of variable quality and any study would need to ensure that the device used was fully validated. These practical considerations are relevant to MCI/AD where home-delivered and sustained therapy is likely to be required.

Protocols of tVNS delivery varied considerably. As demonstrated in Table 2, there was also a range of detail given in tVNS stimulation parameter reporting, making experimental conditions difficult to reproduce. Even the terminology used to describe tVNS protocols varied, further complicating comparisons between studies. In Dolphin’s publication of their study protocol, they detail specific devices and exact settings for reproducibility. Justification for protocols seemed to lean towards practicality over clinical efficacy. This is understandable at present given the lack of evidence that is available about the optimal intensity, duration, and frequency of tVNS delivery. Future studies should look to address this and establish the stimulation settings and time periods that are most efficacious and acceptable.

With this in mind, three of these studies were mechanistic studies considering evidence of target engagement. Particularly with auricular tVNS and new devices, there is some concern about the variability and degree of stimulation achieved and these VSEP and MRI studies highlight both the need for and also the difficulty in confirming this. These tests are complex, sensitive and require very specialist interpretation and so are unlikely to be practical in large high-power longitudinal studies but could potentially play a key role in pilot studies to ensure the proposed protocol is effective in achieving target stimulation prior to conducting larger studies. These and other biomarkers are further explored below.

There was limited cognitive outcome data across these six studies and little consistency between measures used. The positive results by Wang et al. using the global score of MOCA-B are the most significant as MOCA-B is a well-recognised score with availability in different languages and relatively easy to train in and apply. Similarly, although harder to interpret, Merill et al.’s results in MMSE and ADAS-Cog also showed some suggestion of treatment effect. Dolphin et al. used RBANs which is also a well-known tool used in other studies. The use of scores such as ADAS-Cog and the Clinical Dementia Rating Scale, which are most often used in larger dementia trials, is significant. Whilst there may be concerns over the sensitivity of these scores, it could be argued that if tVNS is to be considered as a treatment, then it should be held to the standard of other dementia therapies. Sub-testing of individual cognitive domains varied considerably with nine different tools. Studies reported significant but modest improvements in verbal learning, face–name associative memory testing and 3D navigation [23], but not other domains such as processing and executive function, although the available data is very limited and these areas of cognition have had positive results in other tVNS studies. Aggregating domains in global cognitive scores may avoid “missing” a treatment effect of a therapy that plausibly effects whole brain cognition. Careful consideration is needed for which assessments should be selected particularly considering the time frame between tests and those more vulnerable to a learning bias through repeat measurement.

Given the small number of studies included in this review, it would also be helpful to discuss where cognition has been considered in VNS studies across different subject groups and how these groups might inform future study protocols.

### 4.1. Animal Studies

There are a small number of animal studies that support the mechanism of action for VNS in AD.

Kalinin et al. and Heneka et al. both demonstrated the role of the LC:NA pathway in an AD mouse model. Damage to the LC and reduction in NA increased amyloid burden and reduced microglial phagocytosis of Aβ. Conversely, increases in NA reduced amyloid aggregation and astrocyte activation and promoted neuroprotective markers. [10,26,27]. As VNS acts through the LC to stimulate this pathway, these studies support its protective mechanism.

Considering VNS as an intervention in animals, Cai et al. demonstrated reduced tau and amyloid burden in a post-operative cognitive model and Kaczmarczyk et al. found VNS promoted protective microglial morphology in both an elderly and AD mouse model not seen in young controls [28,29]. Another mechanism of action is suggested in a mouse study that demonstrated VNS protected against blood–brain barrier dysfunction in a traumatic brain injury model as shown by reduced Aquaporin 4 [30].

Considering results from cognitive testing in animal models, by their nature, many studies focus on spatial and working memory with fewer and less definitive results for retention. This may represent the true cognitive effect of VNS or could be due to difficulty testing these memory domains in animals compared to the multidomain testing that is standard in human studies. Results in healthy rats have been mixed [28,31,32]. Greater promise is seen in vascular, neuroinflammatory and intellectual disability models with both improved circulation and recognition and working memory [33,34,35]. A small number of VNS interventions specifically in AD models have shown mixed results. Yu et al. found improved spatial memory but not recognition memory, whilst Yesiltepe et al. demonstrated improved neuropsychiatric symptoms and recovery of retention memory [36,37]. Kamoga et al. induced hippocampal damage as a surrogate for AD but did not show any benefit in spatial learning following VNS which may support that the mechanism of action is more specific to the LC:NA pathway [38].

### 4.2. Human Studies in Healthy Subjects and Other Pathological Populations

There have been a small number of studies assessing cognition in healthy human subjects, mostly under controlled conditions. Some show no effect [39], and others show modest improvements occasionally in various cognitive domains including verbal [40] and associative memory [41] but more often in executive and processing domains such as word processing, [42] rigidity [43], multitasking [44], and creativity [45,46,47]. This may reflect the limited and focused testing that was performed but is supported by a meta-analysis by Ridgewell et al. which found, from 19 studies, that VNS showed a small effect size (0.21) in overall cognitive function that was statically significant in domains of executive function [48]. Only one study looking at a subset of an older population was found and did not demonstrate any significant benefit of VNS [39].

Building from this, two studies examined memory under stress and sleep deprivation. Findings included improved working memory and multitasking particularly during visuo-spatial tasks [49,50].

Whilst studies in epilepsy have typically considered seizure control as their primary endpoint, a small number have considered its impact on cognition as a secondary outcome or add-on study. Results in people with epilepsy include multiple studies using implantable VNS with the advantage that these studies can investigate a much longer duration of therapy, although the disadvantage is that these are not blinded. They have demonstrated mixed results in general, with some again finding improved executive function [51,52,53] and working memory [54] while others show no improvement or even worsening verbal fluency [51,55]. Studies reporting verbal memory and recall memory have also produced mixed results [32,39,52,55,56].

A comprehensive review by Lam et al. considered 29 studies in epilepsy and VNS. Of 19 longitudinal studies, half did not show any change, 8 showed modest improvements in at least one cognitive domain and one study even showed decline. Of 10 acute interventional studies, 4 showed improvement across several cognitive domains but 3 reported a decline. They suggest this could be due to differences in cognitive tests and reduced sensitivity of these longer studies [15].

Similarly in depression, implanted long-term VNS has been an intervention for some time. One study found improved processing and executive function at 10 weeks [57], whilst another demonstrated improved cognitive function most notably in verbal, immediate and delayed recall but also in visuospatial and executive function. This appeared at one month independent of improvements in mood and was preserved at 2 years, arguing against the concerns of “wearing off” over time [58].

More recently a series of studies have considered VNS as an intervention in post-operative cognitive impairment and delirium. A promising initial controlled trial in 124 patients found that tVNS pre-induction reduced the frequency of delayed neurocognitive recovery by nearly 20% [59]. There are currently a further three studies underway in this area [60,61,62,63].

To our knowledge there are no human studies in VNS with a paired intervention. Paired interventions with physical rehab have been shown to improve efficacy in animal and human stroke models [63,64]. One human study is planned to pair tVNS with transcranial magnetic stimulation as a “top-down”, “bottom-up” approach [65]. Interestingly Farmer et al. propose in their review that a paired focused task may improve the efficacy of VNS by ensuring the subject is primed into a specific “brain state” prior to VNS. This is supported by Clark’s early study protocol whereby participants were primed with a verbal learning task followed by VNS which then improved their memory consolidation [3,32,56].

### 4.3. VNS Protocols

A consensus on protocol is yet to be reached in tVNS. A review of protocols by Farmer et al. of 20 long-term and 75 short-term studies highlighted variability in protocols and in particular the level of detail in protocol reporting [3].

When considering the site of tVNS, the conchae is the most commonly stimulated followed by the tragus, and for the sham site, it is the scapha followed by the earlobe [3]. An important paper by Yakinima et al. found stimulation of the cymba conchae alone produced statistically significant activation of the vagally connected area of the brain compared to other sites [66].

Across studies in the Farmer review, VNS intensities vary from 130 µA to 10mA with the most popular range being 4–6 mA. Importantly, whilst it would seem intuitive that a higher intensity of stimulation would produce a greater effect, animal models demonstrate a more complex “therapeutic range”. Increasing intensity does correlate to higher NE levels and greater recruitment of neurons whilst lower levels do not reach a threshold for activity. However, excessive stimulation intensities can switch to an inhibitory effect. These studies are mostly invasive animal studies so cannot be used as a guidance for these “on” and “off” thresholds in human tVNS studies, but these findings emphasise the need for future human studies to consider the use of biomarkers of tVNS activity to confirm an effect in the relevant neural networks for the settings used [13].

There is less variability in frequency with most using a 20–30 Hz range.

Session times vary most of all with at least 14 different regimes identified in the Farmer review ranging from just 15 min a day to 9 h a day with single sessions up to 3 h. The most popular protocol maybe balancing practicality with treatment efficiency is 30 min twice a day. Longitudinal studies range from 10 days to 12 months but many studies are single-intervention only [3].

Reporting on compliance is also often lacking and future longitudinal studies should ensure both measures to check compliance adherence and full reporting of it [3,13].

### 4.4. Biomarkers

The role of biomarkers appears to be essential to enhance VNS delivery and can be divided into two main roles: as a measure of VNS activity itself (demonstrating target engagement) and the longer-term effect of VNS on biomarkers associated with disease severity. Early observed biomarkers can serve as surrogate endpoints in clinical trials and reduce the sample size and cost of studies.

Studies that have investigated markers of VNS activity have considered physiological, imaging and electrophysiological studies such as fMRI, EEG and evoked potentials. These can be particularly helpful as they can demonstrate causality and a dose-based response between parameters of VNS, observed activity, and impact on cognition.

Proposed physiological markers to confirm tVNS activity include heart rate variability and pupil dilation but neither has shown any consistent results across studies [67,68]. Salivary amylase has shown some promise, although again not consistently, and has largely been explored in healthy individuals. P300 or P3b refers to a signal in the event-related potential recorded by electrodes on the scalp. It has gained some interest as a measure of tVNS activity and a surrogate marker for activation of the LC-NA system. Unfortunately, studies have demonstrated variability in its reliability as a biomarker, although it has not yet been explored in an AD/MCI population [14].

In this review, three of the five studies looked at MCI alone and not AD. This targets a spectrum of illness of which a subpopulation progresses to AD. However, it is possible that where impairment is milder, treatment effect is harder to detect and possibly harder still given a significant proportion of patients do not have AD pathology or progress to AD and so are likely to reflect a different brain microenvironment [66]. Stratification of patients according to plasma biomarker status is likely to be essential in an adequately powered efficacy trial.

Plasma markers relating to AD pathology have tended to focus on Aβ and tau biomarkers. Clinical trials have largely used invasive CSF testing and expensive PET imaging. Only small amounts cross the BBB so whilst significant progress has been made in developing more sensitive serum assays for peripheral sampling, these are more vulnerable to variability from both intrinsic and laboratory factors. The use of neuronal derived extracellular vesicles (NEVs) may well improve the sensitivity but these studies are still in their infancy [69].

An area of increasing interest particularly in VNS is more general markers of both neural and systemic inflammation. Animal studies have demonstrated changes in various markers within the brain but data on human peripheral markers is more limited. TNF-α, IL-6, IL-10 and Brain-Derived Neurotrophic Factor (BDNF) have been most explored with variable results particularly across the various interleukins. Two studies found BDNF increases within the brain in an AD and healthy rodent model following VNS [27,70], and two found VNS reduced serum TNF-alpha, although neither were AD-specific [7,35,71].

In addition to various inflammatory markers, some studies have considered markers of BBB function such as Aquaporin-4 [30] or more general big data markers such as epigenetics and proteomics [27,72].

Two of the studies in this review considered vagus somatosensory evoked potentials (VSEPs). Metzger et al. found increasing latency across healthy subjects to MCI to AD and Polak found increased latencies between AD and healthy controls that was not seen between depression and healthy controls, suggesting VSEP latency reflects Alzheimer’s pathology more specifically. Whilst the Metzger study would suggest VSEPs as a potential biomarker for pre-clinical AD, the larger Polak study found the overall predictive power VSEPs was only 66% with no significant difference between healthy controls and those with milder AD. However, the potential of this non-invasive relatively cost-efficient approach certainly warrants further study [22,23].

## 5. Limitations

The main limitation of this targeted review is the very small and heterogeneous evidence base: only six interventional studies in MCI/AD are included, with diverse populations, devices, stimulation parameters, and outcome measures, which precludes any robust pooled estimate of efficacy. Most studies are early-phase, underpowered, and designed around feasibility, safety, or mechanistic biomarkers rather than clinically meaningful cognitive or functional endpoints, limiting conclusions about treatment effect. Diagnostic approaches also vary, with limited use of AD-specific biomarkers or advanced imaging, so underlying pathology is not uniform and findings may not generalise to biomarker-confirmed AD populations. The narrative synthesis, while appropriate to the heterogeneity, introduces subjectivity and cannot address publication bias or small-study effects. Finally, the search strategy, although broad, is restricted to selected databases and may miss non-indexed, non-English, or unpublished negative studies, potentially overestimating the apparent promise of VNS in this population.

## 6. Conclusions

This literature review demonstrates the very limited evidence base for human VNS in MCI and AD. Studies are variable in design, patient cohort, intervention and measures. There is some evidence to support that non-invasive tVNS is well tolerated and that participants with MCI and mild AD could apply tVNS at home for a prolonged period. The use of global cognitive scores may be sensitive to detect treatment effects, while sub-analysis of specific domains within these scores such as recall memory, executive function and visuospatial working memory may help specify tVNS effects further. Additional measures of functional ability and quality of life should be considered as important patient outcomes and have been little explored in these or other VNS studies. Study design should consider measurement of patient compliance and mechanistic biomarkers of VNS for validation of treatment effect. Studies reporting the participant experience of using tVNS in MCI and AD are also lacking and will help inform optimal compliance going forward. Other recommendations would be the use of plasma biomarkers for defining the MCI population and considering these and other markers of inflammation as outcome measures. Full reporting of VNS device settings and protocols will enhance re-producibility and comparability between studies. Addition of a cognitive training tool may be helpful to both promote compliance and reinforce treatment effect.

## Figures and Tables

**Table 1:** Overview of human interventional studies: study design and bias.

Study	Population	Number of Participants	Design	Blinding	Bias (MMAT)
The efficacy and safety of transcutaneous auricular vagus nerve stimulation in patients with mild cognitive impairment: A double blinded randomised controlled study. Wang et al. 2022 (China) [20]	MCI	60 (30 VNS, 30 sham)	Randomised placebo-controlled study	Double	3/5
Improvements in associative memory and spatial navigation with acute transcutaneous Vagus Nerve Stimulation in Mild Cognitive Impairment: preliminary data. Dolphin et al. 2023 (Ireland) [24]	MCI	28	Placebo-controlled crossover study	Single	4/5
The Effects of Transcutaneous Vagus Nerve Stimulation on Functional Connectivity Within Semantic and Hippocampal Networks in Mild Cognitive Impairment. Murphy et al. 2023 (US) [21]	MCI	50 (25 VNS, 25 sham)	Randomised controlled single-intervention study	Single	5/5
Vagus Somatosensory Evoked Potentials (VSEPs) A Possibility for Diagnostic Improvement in Patients with Mild Cognitive Impairment? Metzger et al. 2012 (Germany) [23]	AD, MCI, healthy controls (HCs)	37 (8 AD, 6 MCI, 23 HCs)	Non-randomised cross-sectional controlled study	None	4/5
Vagus nerve stimulation in patients with Alzheimer’s disease: additional follow-up results of a pilot study through 1 year. Merrill et al. 2006 (Sweden) [25]	AD	17	Non-randomised interventional self-controlled study	Single	5/5
Vagus somatosensory evoked potentials are delayed in Alzheimer’s disease, but not in major depression. Polak et al. 2014 (Germany) [22]	AD, healthy controls, major depression (MD)	224 (two age-matched groups: 55 AD + 55 HCs; 57 MD, 55 HCs)	Non-randomised prospective interventional study with age-matched controls	None	5/5

**Table 2:** Breakdown of VNS protocols.

Study	Type of VNS	Site	Frequency	Current	Duration of VNS	Duration of Study	Tolerability
Wang 2022 [20]	TCA	Bilateral Conchae (CO15, CO10) Sham: Helix	(20 Hz: 10 s, 100 Hz: 50 s) Per minute	0.6–1 mA as per patient tolerance	30 min, twice a day, 5/7 days per week	24 weeks	Well tolerated. One withdrawal (tinnitus, sore throat, tooth ache).
Dolphin 2023 [24]	TCA	Left Conchae Sham: Left Earlobe	8 Hz	0.1–5 mA to comfort (mean tVNS: 2.5, sham VNS: 2)	30 s on, 30 s off throughout 21.2 min mean total	Single visits (tVNS and sham, 7–10 d washout)	Not published.
Murphy 2023 [21]	TCA	Left Tragus Sham: Left Earlobe	20 Hz	80% max tolerance (up to 10 mA); mean VNS: 7.3, sham: 7.7	Approx 6 min	Single visit	Discomfort 1–10 scale. VNS: 1.6. Sham: 1.5. No withdrawal.
Metzger 2012 [23]	TCA	Left and Right Tragus	0.5 Hz	8 mA	0.1 ms alternating ears (100 cycles per ear for analysis)	Single visit	Not recorded.
Merrill 2006 [25]	Invasive VNS	NA	20 Hz	Up to 0.75 mA as tolerated (1 patient reduced to 0.5 mA)	30 s on, 5 min off; continuous	1 year	1 participant needed adjustment of settings due to pain and hoarseness. Hoarseness/cough most common; mild–moderate; resolved over time.
Polak 2014 [22]	TCA	Right Tragus	Not recorded	8 mA	0.1 ms	Single visit	Not recorded.

TCA: Transcutaneous Auricula.

**Table 3:** Results of cognitive outcome measurements.

Study	Cognitive Measure	Domain of Cognition	Outcomes of VNS Intervention
Wang 2022 [20]	MOCA-B: Montreal Cognitive Assessment Basic	Global (no breakdown)	Significant improvement post intervention between arms (*p* = 0.033)
	AVLT-H: Auditory Verbal Learning Test-Huashan Version	Memory: Immediate and delayed recall	No statistical difference between arms post intervention Significant difference from baseline in tVNS arm not sham arm Statistical difference between endpoints between arms
	SST A&B: Shape Trails Test	Executive function	No significant difference
	Animal Fluency Test	Verbal fluency	Unable to analyse (statistically different between arms at baseline)
	Boston Naming Test	Word recall/semantic	Significant increase in both active and sham groups with no significant group-level differences
Dolphin 2023 [24]	Face Name Association Task (FNART)	Associative memory (name recall and recognition)	Improved recall accuracy only following tVNS (70% tVNS and 45% baseline (*p* = 0.016), 50% sham (*p* = 0.021))
	Sustained Attention Response Time	Attention	Nil significant
	Sea Hero Quest	3D navigation	Improved navigation in active and sham groups (improved 12.6 s to baseline (*p* = 0.02), 13 s to sham (*p* = 0.00))
	RBANS Battery: Language: List Learning/Recognition/Recall, Semantic Fluency	Language	Nil significant
	RBANS Battery: Working Memory: Digit Span Forwards & Backwards	Working memory	Nil significant
Murphy 2023 [21]	Nil Formal. Y/N Subjective Question To Change In Thinking Memory	Memory	VNS (Y), 6/25 sham (Y), 9/25 not significant
Metzger 2012 [23]	Not Measured	Not measured	Not measured
Merrill 2006 [25]	ADAS-Cog	Global (no breakdown)	At 6 months 13 of 17 participants showed no decline or improvement. Median change +3 points (significant *p* = 0.012). At 1 year 7/17 showed no decline or improvement. Median change −2 points (not significant)
	MMSE	Global (no breakdown)	At 6 months 12 of 17 participants showed no decline or improvement. Median change +2 points (significant *p* = 0.015). At 1 year 12/17 showed no decline or improvement. Median change +2 points (not significant)
Polak 2014 [22]	Not Measured	Not measured	Not measured

**Table 4:** Ongoing studies on VNS in older individuals or cognitive impairment.

Study Name	Population	Design	Intervention	Cognitive Measure	Biomarker
NCT04908358 (USA)	Older adults	1. Randomised double-blind crossover; 2. placebo-controlled	tVNS for 10 sessions of 10 min over 2 weeks	Face–name association memory task	IL-1β, IL-2, IL-6, IL-8, TNF-α, MIP1B, MCP-1, C1q, C3, TREM2. fMRI
NCT06923007 (China)	Alzheimer’s disease	Randomised double-blind placebo-controlled	tVNS for 6 months, 30 Hz and 250 μs, 0.8 mA	ADAS-Cog Subscale; MMSE; AVLT; CDR: Sum of Boxes; CDR: Global Score; AD Cooperative Study: ADLs	Possible additional of blood/CSF sampling, MRI, EEG and PET
NCT07214194 (USA)	Older adults with young controls	Randomised single-blind placebo-controlled within subjects	tVNS to cymba conchae stimulation during memory task with 30 trials per phase	Change in Recognition Memory (d-prime)	Pre-clinical Alzheimer’s disease pathology (pTau217, pTau181, Aβ42:40). Gut–brain axis measures
(China) NCT06923007	Mild–moderate AD	Randomised single-blinded placebo-controlled trial	iVNS	ADAS-Cog, MMSE, CDR, AVLT, Digit Span Test, Trail Making Test, Boston Naming Test, Clock drawing test, Global Deterioration Scale	fMRI, event-related potential (EEG), norepinephrine transporter PET
NCT05514756# (Ireland)	MCI	Randomised single-blinded crossover	tVNS cymba conchae, 8 Hz up to 60 min	Face Name Association Test, SART, Sea Hero Quest, RBANS battery, Digit Span Test	Near-infrared spectroscopy, heart rate, IL-1RA, IL-6, IL-8, IL-10, IL-17 A, MIP-1 β, IL12p70, IP-10, TNF-α, MCP-1, exotaxin, IFN-γ
NCT04877782 (Netherlands)	Older adults	Pseudo-randomised single-blind crossover	tVNS cymba conchae	Face–name association task	fMRI

## Data Availability

No new data were created or analysed in this study. Data sharing is not applicable to this article.

## Abbreviations

The following abbreviations are used in this manuscript:

AD	Alzheimer’s Disease
ADAS-Cog	Alzheimer’s Disease Assessment Scale-Cognitive Subscale
ChEI	Cholinesterase Inhibitor
CSF	Cerebrospinal Fluid
fMRI	Functional Magnetic Resonance Imaging
LC	Locus Coeruleus
MCI	Mild Cognitive Impairment
MMAT	Mixed Methods Appraisal Tool
MMSE	Mini-Mental State Examination
MOCA	Montreal Cognitive Assessment
NA	Noradrenaline
RCT	Randomised Controlled Trial
TCA	Transcutaneous Auricular
TNF-α	Tumour Necrosis Factor Alpha
tVNS	Transcutaneous Vagus Nerve Stimulation
VSEP	Vagus Somatosensory Evoked Potential
